# Amphibian (*Xenopus laevis*) Interleukin-8 (CXCL8): A Perspective on the Evolutionary Divergence of Granulocyte Chemotaxis

**DOI:** 10.3389/fimmu.2018.02058

**Published:** 2018-09-12

**Authors:** Daphne V. Koubourli, Amulya Yaparla, Milan Popovic, Leon Grayfer

**Affiliations:** Department of Biological Sciences, George Washington University, Washington, DC, United States

**Keywords:** interleukin-8, amphibian, granulocyte, chemotaxis, CXCL8, FV3

## Abstract

The glutamic acid-leucine-arginine (ELR) motif is a hallmark feature shared by mammalian inflammatory CXC chemokines such the granulocyte chemo-attractant CXCL8 (interleukin-8, IL-8). By contrast, most teleost fish inflammatory chemokines lack this motif. Interestingly, the amphibian *Xenopus laevis* encodes multiple isoforms of CXCL8, one of which (CXCL8a) possesses an ELR motif, while another (CXCL8b) does not. These CXCL8 isoforms exhibit distinct expression patterns during frog development and following immune challenge of animals and primary myeloid cultures. To define potential functional differences between these *X. laevis* CXCL8 chemokines, we produced them in recombinant form (rCXCL8a and rCXCL8b) and performed dose-response chemotaxis assays. Our results indicate that compared to rCXCL8b, rCXCL8a is a significantly more potent chemo-attractant of *in vivo*-derived tadpole granulocytes and of *in vitro*-differentiated frog bone marrow granulocytes. The mammalian CXCL8 mediates its effects through two distinct chemokine receptors, CXCR1 and CXCR2 and our pharmacological inhibition of these receptors in frog granulocytes indicates that the *X. laevis* CXCL8a and CXCL8b both chemoattract tadpole and adult frog granulocytes by engaging CXCR1 and CXCR2. To delineate which frog cells are recruited by CXCL8a and CXCL8b *in vivo*, we injected tadpoles and adult frogs intraperitoneally with rCXCL8a or rCXCL8b and recovered the accumulated cells by lavage. Our transcriptional and cytological analyses of these tadpole and adult frog peritoneal exudates indicate that they are comprised predominantly of granulocytes. Interestingly, the granulocytes recruited into the tadpole, but not adult frog peritonea by rCXCL8b, express significantly greater levels of several pan immunosuppressive genes.

## Introduction

The mammalian cysteine-X-cysteine (CXC) chemokines can be subdivided into two groups depending on whether they posses the Glu-Leu-Arg (ELR) motif at their N-termini (1, 2). This ELR motif is responsible for binding of these chemokines to their cognate receptors on neutrophils, resulting in the neutrophil chemotaxis. By contrast, the CXC chemokines lacking this motif do not attract neutrophils and instead target mononuclear phagocytes and different lymphocyte subsets (1, 2). With the exception of *Gadiformes* (cod, haddock), the teleost fish CXC chemokines lack ELR motifs and instead generally possess X(other residue)-Leu-Arg (XLR, DLR in salmonids) motifs, which are presently thought not to contribute to the function of these respective CXC chemokines or their recruitment of fish neutrophils (3). Because the mutation of the mammalian CXC chemokine ELR motifs to DLR did not abolish their neutrophil recruitment, it was originally thought that the fish DLR (and XLR) motifs function as the mammalian CXC chemokine ELR counterparts (3). However, more recent work has demonstrated that the fish CXC chemokine DLR/XLR motifs are dispensable to the fish neutrophil chemotaxis (4).

CXCL8 (interleukin-8, IL-8) is an important inflammatory CXC chemokine, first discovered in mammals for its role in the chemotaxis of neutrophils (5). CXCL8-mediated neutrophil recruitment occurs as the result of this chemokine binding to the G protein-coupled CXC chemokine receptor 1 (CXCR1, CXCL8Rα) or CXCR2 (CXCL8Rβ) (6, 7). Of these CXC chemokine receptors, CXCR1 is only ligated by CXCL8 and CXCL6, whereas CXCR2 is bound by several chemokines including CXCL8, CXCL1, and CXCL2 (6, 7). CXCL8 is important to both the initiation as well as the resolution of inflammatory responses. By recruiting neutrophils to sites of injury and/or infection, this chemokine promotes the resolution of tissue damage and clean up of infiltrating pathogens through neutrophil-mediated phagocytosis, respiratory burst, and the release of neutrophil extracellular traps (8). In turn, CXCL8 facilitates wound repair by activating the angiogenic response and by eliciting endothelial cell proliferation, survival, and recruitment (9), resulting in the formation of new blood vessels (10), thereby contributing to the resolution of inflammatory stimuli and promoting healing.

*Cxcl8* genes have been identified across a range of bony fish species, with many species encoding multiple CXCL8 isoforms. Cyprinid fish such as zebrafish and carp encode two distinct *Cxcl8* genes (11, 12) corresponding to two *Cxcl8* homologs that have been designated as *Cxcl8_L1* and *Cxcl8_L2*. The expression of both fish *Cxcl8_L1* and *Cxcl8_L2* genes is upregulated in response to bacterial infection (13) and wound-associated inflammation (12). Interestingly, under certain immune conditions, these two genes are differentially regulated (14, 15). Despite lacking ELR motifs, both of these cyprinid CXCL8 homologs chemoattract fish neutrophils (11, 12). While salmonid fish such as trout encode several *Cxcl8* genes (16), these all share close homology to the cyprinid *Cxcl8*_*L1* [76] and the trout CXCL8 likewise chemoattracts fish neutrophils (17). Indeed, while *Cxcl8* chemokines have been identified across multiple teleost fish species, as mentioned above, all of these *Cxcl8* genes lack the ELR motif, with the exception of *Gadiformes* such as Atlantic cod (18) and haddock (19).

Here we report on the amphibian (*Xenopus laevis*) *Cxcl8* isoforms (designated *Cxcl8a* and *Cxcl8b*), only one of which possesses an ELR motif. We show that these frog *Cxcl8a* and *Cxcl8b* genes are differentially expressed in healthy animals and following immunological challenge. Moreover, we demonstrate that these frog CXCL8 chemokines possess distinct chemoattractive capacities and may have functionally diverged to recruit distinct tadpole granulocyte populations.

## Materials and methods

### Animals, culture media, and conditions

Outbred tadpole and adult *X. laevis* were purchased from the Xenopus 1 facility, housed and handled under strict laboratory and IACUC regulations (Approval number 15-024).

The cell culture media and conditions have been previously described (20).

### Production of recombinant frog cytokines and chemokines

The production of recombinant G-CSF, M-CSF, and IL-34 has been previously described (20, 21). The recombinant CXCL8a and CXCL8b were generated by PCR-amplifying the respective sequences, corresponding to the signal peptide-cleaved *Cxcl8a* and *Cxcl8b* transcripts, ligating these into the pMIB/V5 His A insect expression vectors (Invitrogen) and introducing positive clones into Sf9 insect cells (Cellfectin II, Invitrogen). The production of recombinant (r)CXCL8a and rCXCL8b by the transfected Sf9 cells was confirmed by western blot against the V5 epitopes on the recombinants and the positive transfectants were selected using 10 μg/mL blasticidin. These protein expressing cultures were scaled up to 500 ml, grown for 5 days, pelleted and the supernatants collected. The supernatants were dialyzed overnight at 4°C against 150 mM sodium phosphate, concentrated against polyethylene glycol flakes (8 kDa) at 4°C, dialyzed overnight at 4°C against 150 mM sodium phosphate and passed through Ni-NTA agarose columns (Qiagen). Columns were washed with 2 × 10 volumes of high stringency wash buffer (0.5% Tween 20; 50 mM Sodium Phosphate; 500 mM Sodium Chloride; 100 mM Imidazole) and 5 × 10 volumes of low stringency wash buffer (as above, but with 40 mM Imidazole). Recombinant cytokines were eluted using 250 mM imidazole. The eluted recombinant (r)CXCL8a and rCXCL8b were resolved by SDS PAGE, transferred onto nitrocellulose membranes and western blots were performed using an HRP-conjugated mouse anti-V5 (Sigma) to determine which elution fractions contained rCXCL8a (15 kDa) and rCXCL8b (16 kDa). The fractions containing the respective recombinants (Supplemental Figure 1) were pooled, concentrated against polyethylene glycol flakes (8 kDa) at 4°C, dialyzed overnight against saline at 4°C and the protein concentrations were determined by Bradford protein assays (BioRad). Halt protease inhibitor cocktail (containing AEBSF, aprotinin, bestatin, E-64, leupeptin, and pepstatin A; Thermo Scientific) was added to the purified proteins, which were then stored at −20°C in aliquots until use.

### *X. laevis* myeloid cell isolation and culture

Tadpole granulocytes were generated as previously described (20). Briefly, tadpoles (stage NF 54) were injected ip with 1 μg total of rG-CSF using finely pulled glass needles. One day following injection, peritoneal leukocytes were lavaged with saline, enumerated via a hemocytometer and using trypan blue (Sigma) exclusion.

Tadpole and adult frog rCXCL8a- and rCXCL8b-elicited leukocytes were derived by injecting tadpoles (stage NF 54, *N* = 6) and adult frogs (1 year old *N* = 6) with 1 μg/g of body weight of either chemokine. After 4 h of injection, tadpoles, and adults were lavaged with saline and the recovered cells were enumerated by hemocytometer counts, using trypan blue (Sigma) exclusion. The results depicted in Figure 6, corresponding to the transcriptional analysis of these cell populations, are representative of 3 independent experiments, with each iteration performed with cells from 6 tadpoles or adult frogs.

The generation of adult frog M-CSF- and IL-34- macrophages and G-CSF-granulocytes has been previously described (22, 23). Briefly, *X. laevis* adult frogs were sacrificed and their femur bone marrow cells were isolated and incubated with 250 ng/ml of the respective recombinant growth factors at 27°C and 5% CO_2_. After 3 days of culture, the cells were again treated with the respective cytokines and after 5 days of culture, the cells were enumerated and used in the gene expression or chemotaxis assays.

### Frog virus 3 stocks and infections

Frog Virus 3 (FV3) production has been described previously (24). In brief, baby hamster kidney (BHK-21) cells were infected with FV3 (multiplicity of infection; MOI: 0.1), grown at 30°C and 5% CO_2_ for 5 days. The FV3-containing supernatants were collected over 30% sucrose by ultracetrifugation, re-suspended in saline and the viral titers were determined by plaque assay over BHK-21 cells.

Tadpoles (*N* = 5) and adult frogs (*N* = 5) were infected with FV3 by intraperitoneal (ip) injection with 1 × 10^4^ and 5 × 10^6^ PFU of FV3, respectively or mock infected with saline (not containing FV3). Animals were euthanized by tricaine mesylate overdose (tadpoles: 1%; adult frogs: 5%), kidney tissues excised, immediately flash-frozen in Trizol reagent (Invitrogen) over dry ice and stored at −20°C until RNA isolation.

For all *in vitro* infection studies, leukocytes were infected with a multiplicity of infection (MOI) of 0.5 plaque forming units (PFU) of FV3 for 16 h, incubated in the medium described above at 27°C with 5% CO_2_. Subsequently, the cells were trypsinized to remove attached but not internalized virus and washed with saline and processed for RNA isolation and cDNA synthesis. Alternatively, leukocytes were incubated with heat-killed *E. coli* for 16 h prior to RNA isolation and cDNA synthesis.

### Tadpole and adult frog wounding and tissue repair studies

For the tadpole tissue inflammation and repair/regeneration study, 15 tadpoles were anesthetized with tricaine mesylate and the furthermost thirds of their tails were amputated using clean razor blades. Approximately 1 mm sections were cut from the amputated tails (portions closest to the cut site), and used as controls for these studies. After 5 h, 1, 3, and 6 days of the initial amputation, 5 tadpoles (per time point) were anesthetized and ~1 mm sections of their amputated, regenerating tails were excised using clean razor blades. These 1 mm section were used for RNA isolation, as described below.

For the adult frog, wounding and repair experiments, 20 frogs (1 year old) were anesthetized with tricaine mesylate and pieces of skin ~1 mm^2^ were excised from their hind right legs. These sections were used as the expression controls for this study. After 5 h and 1, 3, and 12 days of the initial incisions, 5 of the frogs were again anesthetized and the skin around the initial incision was removed in a 1 mm perimeter, and subjected to RNA isolation.

### RNA isolation, cDNA synthesis, and quantitative gene expression analyses

For all experiments, tadpole and adult frog cells or kidney tissues from FV3-infected animals were homogenized in Trizol reagent (Invitrogen), flash frozen on dry ice and stored at −80°C until RNA isolation in accordance to manufacturer's directions. The isolated RNAs (500 ng total) were reverse transcribed into cDNAs using cDNA qscript supermix (Quanta), according to manufacturer's instructions.

All quantitative analysis of *X. laevis* gene expression was performed using the CFX96 Real-Time System and iTaq Universal SYBR Green Supermix. The BioRad CFX Manager software (SDS) was employed for all expression analysis. All primers were validated prior to use and the sequences of all employed primers are listed in the Supplemental Table 1.

All expression analyses were conducted relative to the *Gapdh* endogenous control gene. The expression of the *Cxcl8a* and *Cxcl8b* genes was directly compared by calculating the delta∧delta CT values for these two genes relative to the highest CT value (lowest mRNA levels) across all of the derived *Cxcl8a* and *Cxcl8b* CT values. Likewise, the expression of *Cxcr1* and *Cxcr2*, as presented in Figures 2, 3, 4C and Supplemental Figure 2B, was directly compared by calculating the delta∧delta CT values, relative to the lowest observed CT value across the derived *Cxcr1* and *Cxcr2* CT values within the respective experiments. For all other gene expression analyses, the delta∧delta CT values were derived relative to the lowest expressing tissue/cell type within the given data set (highest CT) and the derived relative quantification data were normalized against that lowest sample.

### Chemotaxis assays

All chemotaxis assays were performed using blind well chemotaxis (Boyden) chambers (Neuro Probe), with medium alone or 10^3^-10^−7^ ng/ml of rCXCL8a or rCXCL8b (in culture medium) loaded into bottom wells of these chambers. The bottom wells were overlaid with 13 mm chemotaxis filters (5 μm pore size; Neuro Probe) and tadpole or adult frog granulocytes (10^5^ cells/well) were added to the top wells. After 3 h of incubation at 27°C with 5% CO_2_, the top layers were aspirated, the top sides of the filters were wiped with cotton swabs. The filters were then removed, washed, and the bottom faces of the filters were stained with Giemsa stain and the numbers of migrating cells was determined by counting ten random fields of view per filter (40x objective). For the chemokinesis experiments, both the bottom and the top wells of the chemotaxis chambers were loaded with the most potent chemoattractive concentrations of either chemokine; tadpole granulocyte: 10^−3^ ng/ml of rCXCL8a and 10^1^ ng/ml of rCXCL8b; adult granulocytes: 10^−5^ ng/ml of rCXCL8a and 10^−3^ ng/ml of rCXCL8b. The assays were performed as above. The reparixin (CXCR1/2 inhibitor, 1 and 100 nM, MCE) and SB265610 (CXCR2 inhibitor, 5 and 100 nM, Sigma) inhibition studies were carried out by using the respective optimal doses of rCXCL8a or rCXCL8b to examine tadpole and adult granulocyte migration in the presence of 0, 1, or 100 nM final concentrations of reparixin across both lower and upper chambers. Cells from three individual animals (*N* = 3) were used to test each concentration of either rCXCL8. The tadpole and adult frog granulocyte chemotaxis toward the most chemo-attractive concentrations of rCXCL8a and rCXCL8b was confirmed twice, independently, using cells from three individuals for each experiment. The results from the 3 independent studies (*N* = 9) were combined and are presented in Figure 4. The pharmacological inhibition experiments were performed using cells from 4 tadpoles (per treatment group) and 4 adult frogs (*N* = 4).

### Anti-*X. laevis* G-CSFR polyclonal antibody

A recombinant form of a fragment of the extracellular portion of the *X. laevis* G-CSFR was produced by PCR-amplifying the corresponding signal peptide-cleaved *Gcsfr* transcript of 702 nucleotides and cloning it into the pET SUMO prokaryotic expression vector (ThermoFisher). This construct was introduced into One Shot Mach-T1R Chemically Competent *E. coli*, (Invitrogen), plated onto kanamycin (50 μg/ml) LB plates and the resulting colonies were screened by colony-PCR for *Gcsfr*-positive constructs. Positive colonies were grown in LB + kanamycin (50 μg/ml), the plasmids were isolated and sequenced to confirm the presence of in frame 702 nt sequences corresponding to the *X. laevis Gcsfr* fragment. Several of these positive clones were introduced into BL21(DE3) One Shot Chemically Competent *E. coli* (Invitrogen), and pilot IPTG induction studies were performed to determine which clones resulted in the highest protein production and to deduce the optimal recombinant protein induction time. According to these pilot studies, the best rG-CSFR-expressing *E. coli* culture was scaled up into several 500 ml LB + kanamycin (50 μg/ml) cultures, grown for 2 h and induced with IPTG (1 mM final concentration) for an additional 4 h. The cultures were then collected by centrifugation and lysed by 3 repeated freeze-thaw cycles in the presence of B-PER bacterial protein extraction reagent (ThermoScientific). The lysates were then incubated with Ni-NTA agarose (Qiagen) to isolate the (His^6^-tagged) rG-CSFR. The isolation of the rG-CSFR using the Ni-NTA beads was conducted using the same procedure as described above for the rCXCL8a/b isolation. The rG-CSFR elution fractions containing the protein were pooled, concentrated against polyethylene glycol flakes (8 kDa) at 4°C, dialyzed overnight against saline at 4°C and the protein concentration was determined by the Bradford protein assay (BioRad). The isolated rG-CSFR (1 mg total) was submitted for rabbit immunization protocols (ProSci Inc). The resulting rabbit immune sera (2 rabbits) were examined by western blot against the rG-CSFR to determine which of the two sera had greater detection of the recombinant, using secondary HRP-conjugated goat anti-rabbit IgG (ThermoScientific) to for the detection. The corresponding serum was then applied to a HiTrap Protein A HP column (GE Health) to isolate the IgG fraction from the rabbit serum. A Sulfo-Link Protein Kit (ThermoScientific; according to manufacturer's instructions) was then used to purify the IgG fraction that cross-reacted with the rG-CSFR. To confirm the specificity of this reagent, we pre-absorbed this purified anti-rG-CSFR IgG against the rG-CSFR prior to western blot analysis of rG-CSFR. Whereas the non-pre-absorbed antibody detected the rG-CSFR at the expected 40 kDa molecular weight (Supplemental Figure 2A, lane 1), the rGCSFR-pre-absorbed antibody did not (Supplemental Figure 2A, lane 2), confirming the specificity of this reagent.

For staining of rCXCL8a- and rCXCL8b-elicited granulocytes, the recovered cells were cyto-centrifuged onto glass slides, stained with the HiTrap Proetin A HP column and A Sulfo-Link Protein Kit-purified anti-rG-CSFR primary rabbit antibody (2.5 μg total) overnight at 4°C, and goat anti-rabbit IgG Dylight 488 (ThermoScientific) secondary antibodies (1 h) and counterstained with Hoechst nuclear stain (ThermoScientific).

### Statistical analysis

Statistical analysis was conducted using a one-way analysis of variance (ANOVA) and *post-hoc t-*test, using Vassar Stat (http://vassarstats.net/anova1u.html). Probability level of *P* < 0.05 was considered significant.

## Results

### *In silico* analyses of *X. laevis* CXCL8a and CXCL8b

**Figure 1:**
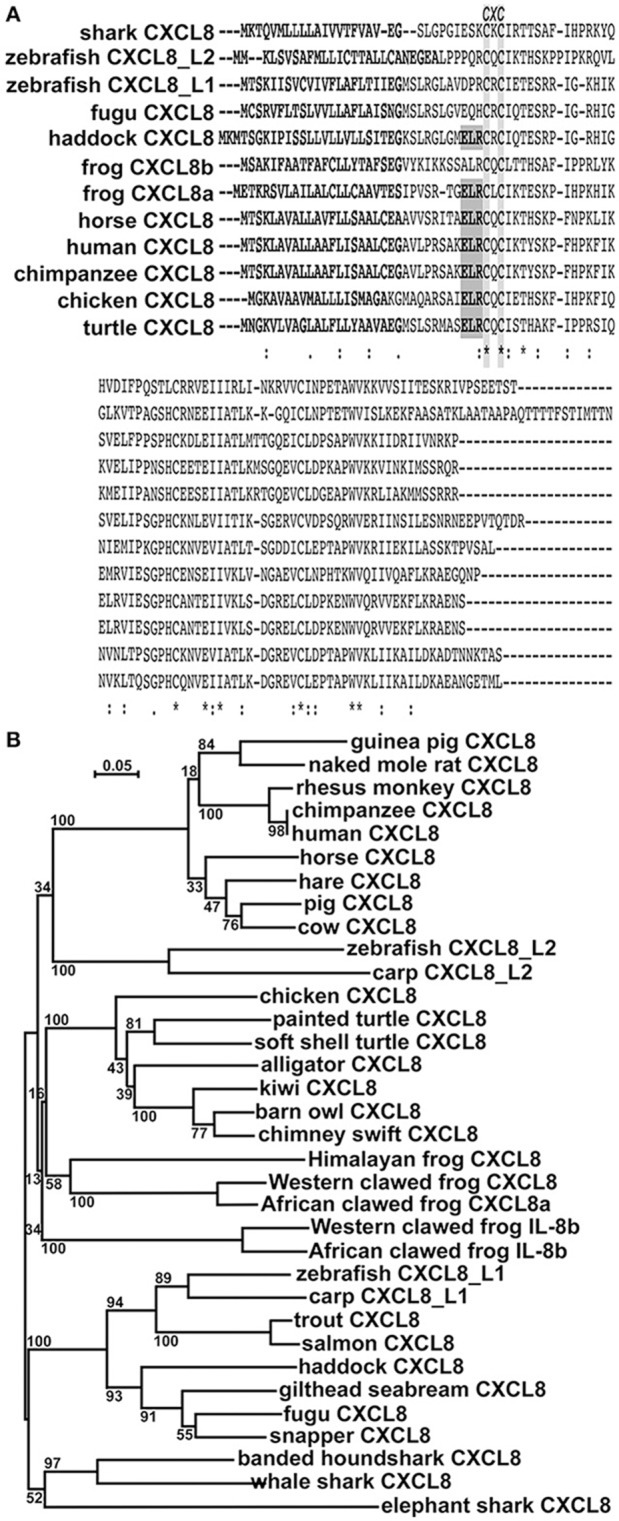
*In silico* analyses of CXCL8 phylogeny and protein sequence conservation.**(A)** The protein alignment was performed using ClustalW2 server. Fully conserved residues are indicated by an asterisk (*), partially conserved and semi-conserved substitutions are represented by “:” and “.”, respectively. Putative signal peptides are bolded, the ELR motif is boxed in gray and the conserved CXC motif is highlighted. **(B)** The phylogenetic tree was constructed using the neighbor joining method and bootstrapped 10,000 times (denoted as %s). The accession numbers for the respective protein sequences are: guinea pig CXCL8: NP_001166870.1; naked mole rat CXCL8: XP_004833980; rhesus monkey CXCL8: NP_001028137.1; chimpanzee CXCL8: NP_000575.1; human CXCL8: NP_000575.1; horse CXCL8: NP_001077420.2; hare CXCL8: ALG04568.1; pig CXCL8: NP_999032.1; cow CXCL8: NP_776350.1; zebrafish CXCL8_L2: XP_009305130.1; carp CXCL8_L2: XP_018936341.1; chicken CXCL8: NP_990349.1; painted turtle CXCL8: XP_005304195.1; soft shelled turtle CXCL8: ACP28489.1; alligator CXCL8: XP_006018817.1; kiwi CXCL8: XP_013807042.1; barn owl CXCL8: XP_009963343.1; chimney swift CXCL8: XP_010001929.1; Himalayan frog CXCL8: XP_018421489.1; Western clawed frog CXCL8a: XP_002942578.2; African clawed frog CXCL8a: OCU00045.1; Western clawed frog CXCL8b: XP_002942578.2; African clawed frog CXCL8b: NP_0010912223.1; zebrafish CXCL8_L1: XP_001342606.2; carp CXCL8_L1: XP_016375461; trout CXCL8: XP_020330727.1; salmon CXCL8: NP_001134182.1; haddock CXCL8: CAD97422.2; gilthead seabream CXCL8: AGS55343.1; fugu CXCL8: NP_001027759.1; snapper CXCL8: AGV99968.1; banded houndshark CXCL8: BAB79448.1; whale shark CXCL8: XP_020370926.1; elephant shark CXCL8: NP_001279539.1.

While all of the vertebrate CXCL8 proteins exhibit the CXC motif, with the exception of haddock (*Gadiformes*), teleost and cartilaginous fish CXCL8 proteins lack the characteristic ELR motif (Figure 1A). Interestingly, while the frog (*X. laevis*) CXCL8a protein possesses an ELR motif, the frog CXCL8b lacks this motif (Figure 1A). Moreover, these *X. laevis* CXCL8a and CXCL8b are fairly different in their respective protein sequences (Figure 1A).

We performed phylogenetic analysis to discern the evolutionary relationships between the amphibian CXCL8 isoforms and the other vertebrate CXCL8 proteins (Figure 1B). The mammalian CXCL8 protein sequences branched as a separate clade from all other vertebrate CXCL8 sequences (Figure 1B). The cyprinid fish CXCL8_L2 sequences formed a separate clade and the avian and reptile CXCL8 sequences together formed a distinct clade (Figure 1B). Notably, the amphibian CXCL8b proteins formed a distinct clade and branched ancestrally to the avian and reptilian CXCL8 clade as well as to the independent amphibian CXCL8a clade. The bony (including the cyprinid CXCL8_L1s) and cartilaginous fish CXCL8 sequences also split into respective clades that branched ancestrally to all other vertebrate CXCL8s (Figure 1B).

### *X. laevis* tadpoles and adult frogs exhibit distinct expression patterns of *Cxcl8a* and *Cxcl8b*

**Figure 2:**
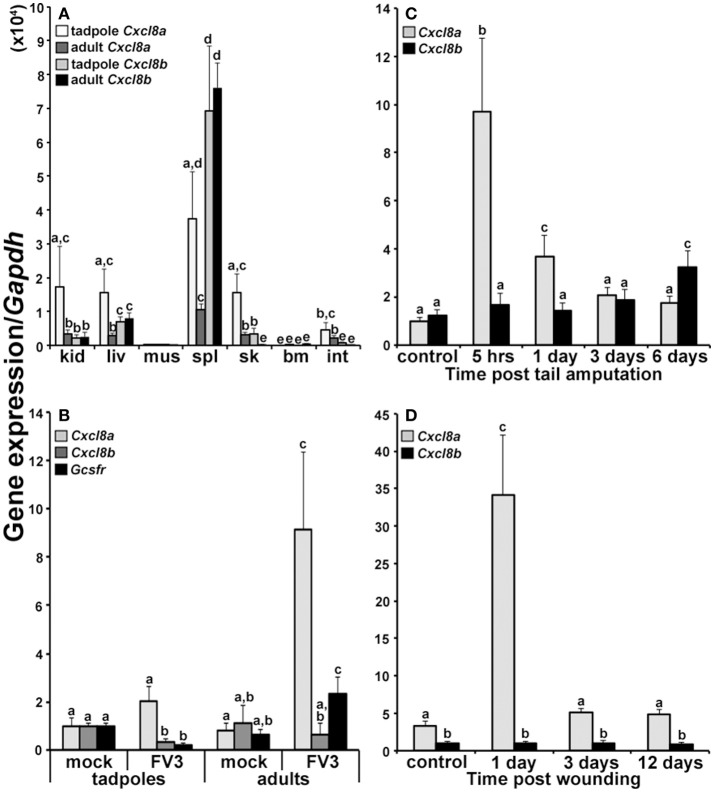
Analyses of *Cxcl8a* and *Cxcl8b* gene expression in healthy and immune challenged animals**. (A)** *Cxcl8a* and *Cxcl8b* gene expression in tadpole and adult kidney (kid), liver (liv), muscle (mus), spleen (spl), skin (sk), bone marrow (bm), and intestine (int) tissues (*N* = 6). **(B)** *Cxcl8a, Cxcl8b, and Gcsfr* gene expression in tadpole and adult kidneys 3 days post FV3 infection (10^4^ PFU/tadpole; 5 × 10^6^ PFU/adult) (*N* = 5). **(C)** *Cxcl8a* and *Cxcl8b* gene expression in amputated tadpole tails (*N* = 5). **(D)** *Cxcl8a* and *Cxcl8b* gene expression in adult frog hind leg skin wounds (*N* = 5). All gene expression was compared relative to *Gapdh* control and all results are presented as means + SEM. Above-head letters denote statistical designations: experimental groups described by distinct letters are statistically different (*P* < 0.05) while those marked by the same letters are not.

We examined the expression of *Cxcl8a* and *Cxcl8b* genes in the tissues of *X. laevis* tadpoles and adult frogs to determine if these two genes are under similar or distinct transcriptional regulation (Figure 2A). Tadpoles possessed significantly greater levels of *Cxcl8a* than *Cxcl8b* mRNAs in their kidney and skin tissues (Figure 2A). Adult frogs possessed significantly greater *Cxcl8b* transcripts in their liver and spleen tissues but greater *Cxcl8a* mRNA levels in their skin and intestines (Figure 2A). Compared to adult frogs, tadpoles possessed significantly greater levels of *Cxcl8a* transcripts in their kidney, skin and intestine tissues (Figure 2A). By contrast, adult frogs exhibited greater splenic expression of *Cxcl8b* than tadpoles (Figure 2A).

### FV3-challenged tadpoles and adult frogs exhibit distinct *Cxcl8a* and *Cxcl8b* gene expression

Anuran (frogs and toads) tadpoles are substantially more susceptible to the Frog Virus 3 ranavirus than the respective adult frogs (25–29). Notably, we recently demonstrated that this susceptibility stems at least in part from the inability of FV3-challenged tadpoles to recruit granulocytes into their kidneys (20), which are a central site of FV3 replication (24). Here we examined the expression of *Cxcl8a* and *Cxcl8b* in the kidneys of tadpoles and adults 3 days after FV3 infection, to discern the potential roles of these chemokines in this granulocyte recruitment (Figure 2B). After 3 days of FV3 infection, tadpoles did not exhibit significant changes in their expression of *Cxcl8a* and possessed significantly decreased gene expression of *Cxcl8b* and the granulocyte colony stimulating factor receptor (*Gcsfr*) granulocyte marker (Figure 2B). By contrast, FV3-infected adult frogs possessed significantly elevated *Cxcl8a* (but not *Cxcl8b*) mRNA levels, concomitant with increased kidney Gcsfr gene expression (Figure 2B), suggesting that *Cxcl8a* (but not *Cxcl8b*) may be involved in the adult frog granulocyte recruitment to this FV3 infection site.

### The frog *Cxcl8a* and *Cxcl8b* genes are differentially expressed during wounding and repair

To further define the potential roles of the frog CXCL8a and CXCL8b under inflammatory and wound repair settings, we examined the expression of *Cxcl8a* and *Cxcl8b* during the amputation and regeneration of tadpole tails and following wounding and repair of adult frog skin tissues (Figures 2C,D, respectively). Tadpoles exhibited elevated *Cxcl8a* but not *Cxcl8b* gene expression 5 h and 1 day post tail clipping (Figure 2C) and adult frogs likewise possessed significantly increased *Cxcl8a* but not *Cxcl8b* mRNA levels 1 day post skin wounding. Six days after the tail clipping, tadpoles had regenerated a substantial proportion of their tails, and this corresponded to significantly elevated *Cxcl8b* (but not *Cxcl8a*) gene expression (Figure 2C). By 12 days post injury, adult frogs skins were completely healed but we did not see changes in the expression of the adult skin *Cxcl8b* at any examined time during the skin wounding/repair study (Figure 2D).

### Frog myeloid cells respond to immune challenge by upregulating *Cxcl8a*

**Figure 3:**
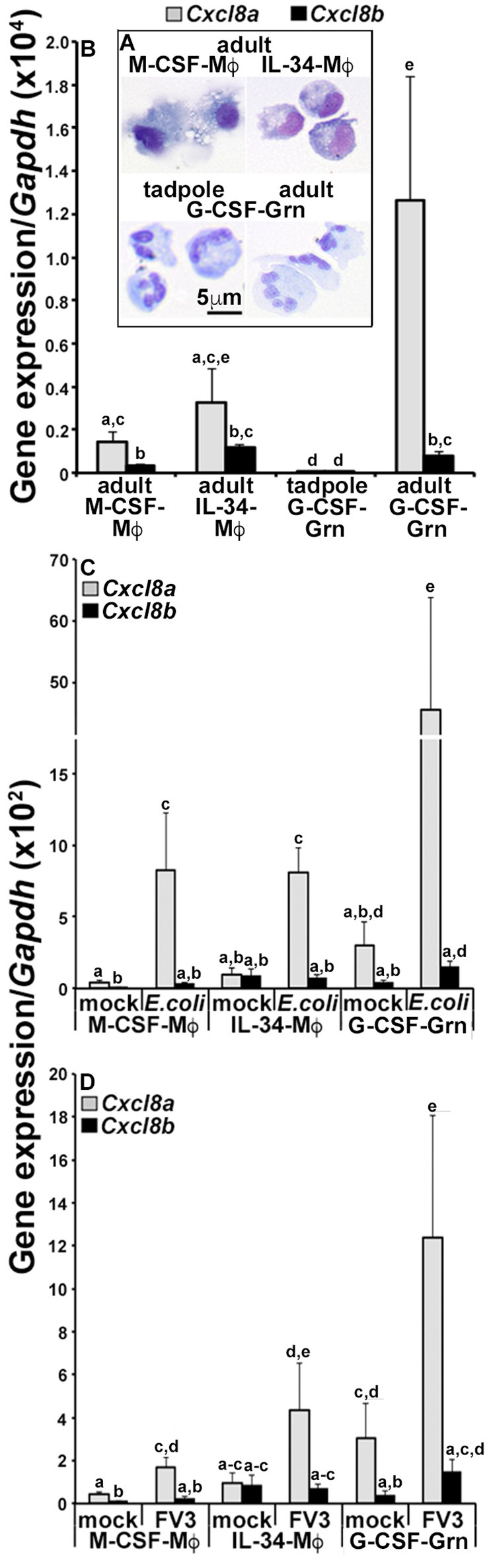
Analyses of *CXCL8a* and *CXCL8b* gene expression in frog macrophages and granulocytes**. (A)** Cytology of tadpole rG-CSF-elicited peritoneal granulocytes (G-CSF-Grn) and adult frog bone marrow-derived macrophages, differentiated with rM-CSF (M-CSF-Mϕ) or rIL-34-Mϕs (L-34-Mϕ) and granulocytes (Grn), differentiated with rG-CSF (G-CSF-Grn). **(B)** The adult frog M-CSF-Mϕ, IL-34-Mϕ, and G-CSF-Grn were examined for their steady state gene expression of *Cxcl8a* and *Cxcl8b*. **(C)** The adult frog M-CSF-Mϕ, IL-34-Mϕ, and G-CSF-Grn were either mock infected (saline) or challenged with FV3 at a multiplicity of infection of 0.5 PFU/cell. After 24 h of challenge, cells were examined for *Cxcl8a* and *Cxcl8b* gene expression relative to *Gapdh* (*N* = 5). **(D)** M-CSF-Mϕ, IL-34-Mϕ, and G-CSF-Gran were mock challenged (saline) or challenged with heat-killed *E. coli* for 24 h before *Cxcl8a* and *Cxcl8b* gene expression analysis, relative to *Gapdh* (*N* = 5). All results are presented as means + SEM. Above-head letters denote statistical designations: experimental groups described by distinct letters are statistically different (*P* < 0.05) while those marked by the same letters are not.

Our *in vivo* expression studies indicated that frogs increased their *Cxcl8a* but not *Cxcl8b* gene expression following immune challenge and under inflammatory settings. We previously demonstrated that the tadpole and the adult frog granulocyte-colony stimulating factor (G-CSF)-differentiated granulocytes (Grn) and the adult frog macrophages (Mϕs) differentiated by the macrophage-colony stimulating factor (M-CSF) and interleukin-34 (IL-34) cytokines are important innate immune effectors of these animals (20, 21). Accordingly, here we examined the expression of *Cxcl8a* and *Cxcl8b* in tadpole rG-CSF-derived peritoneal granulocytes and adult frog bone marrow-derived, M-CSF- or IL-34-Mϕs and G-CSF-granulocytes (Figure 3) We previously demonstrated that these respective cultures comprise predominantly (85–95%) of cells that morphologically represent the respective populations (Figure 3A) and express distinct myeloid markers and immune genes (20, 23).

At steady state, the adult frog G-CSF-granulocytes had the greatest expression of *Cxcl8a*, with comparable *Cxcl8a* transcripts detected in the adult frog M-CSF- and IL-34-Mϕs and much lower *Cxcl8a* mRNA in the tadpole G-CSF-granulocyte (Figure 3B). The adult frog G-CSF-granulocytes, M-CSF- and IL-34-Mϕs exhibited similar *Cxcl8b* expression levels, which were higher than the *Cxcl8b* transcript levels detected in the tadpole G-CSF-granulocytes (Figure 3B). While all of the examined adult frog myeloid populations possessed greater expression of *Cxcl8a* than *Cxcl8b*, the tadpole granulocytes expressed similar levels of both chemokine isoforms (Figure 3B).

Since the tadpole cells exhibited such negligible baseline *Cxcl8a* and *Cxcl8b* expression, we focused on the adult myeloid cell populations to examine their expression of these chemokine genes following *in vitro* challenge with either heat-killed *E. coli* (Figure 3C) or FV3 (Figure 3D). Notably, after challenge with either heat-killed *E. coli* or FV3, all three immune populations upregulated their *Cxcl8a* gene expression (Figures 3A,B). While the *E. coli* and FV3-stimulated G-CSF-granulocytes also upregulated their *Cxcl8b* transcript levels (albeit to a much lesser extent than *Cxcl8a*), the M-CSF- and IL-34-Mϕs did not (Figures 3A,B).

### The frog CXCL8a and CXCL8b possess distinct chemotactic capacities

**Figure 4:**
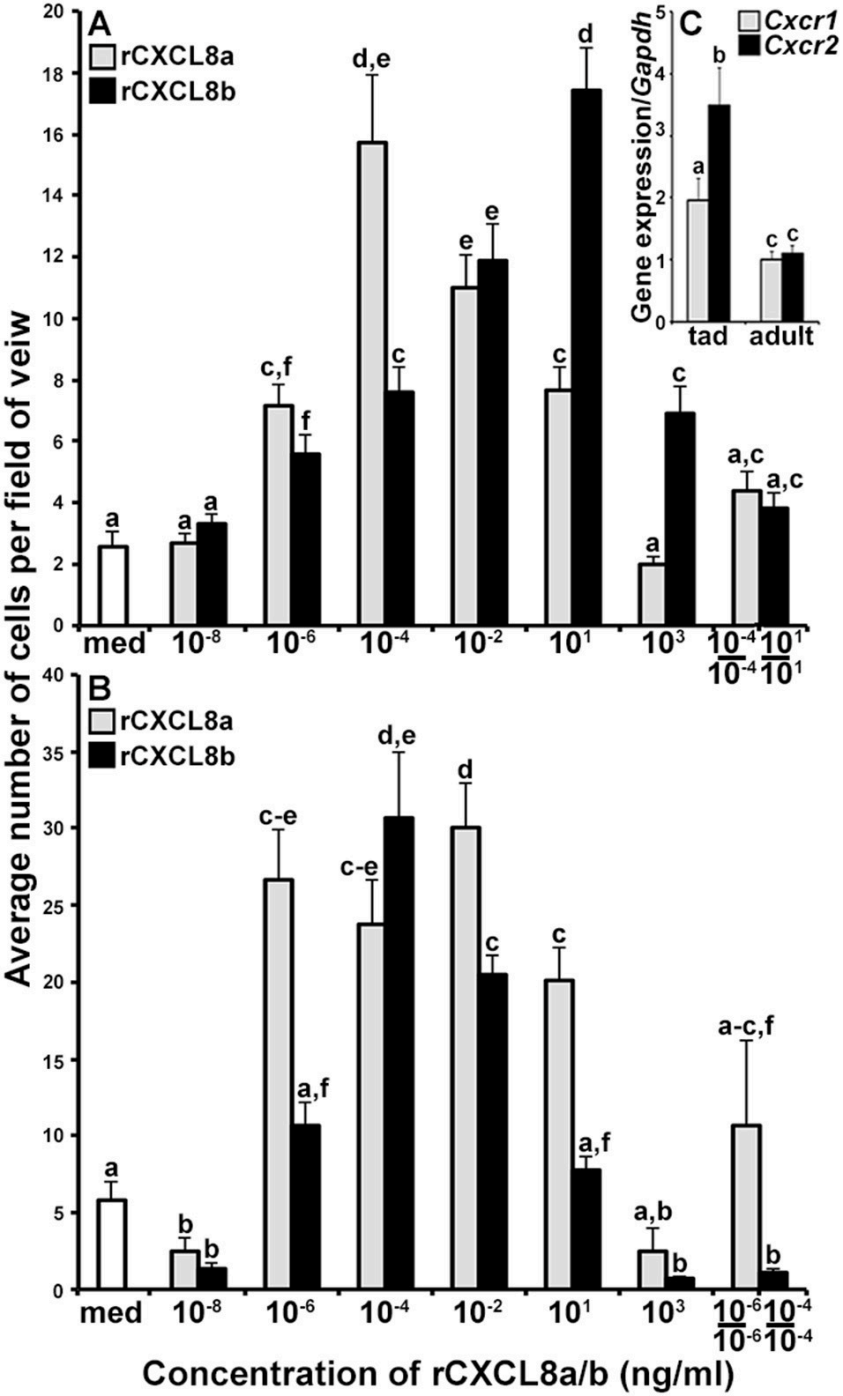
The rCXCL8a and rCXCL8b elicit distinct chemotaxis responses in tadpole and adult granulocytes. **(A,B)** Medium or increasing concentrations of rCXCL8a or rCXCL8b were loaded into bottom chemotaxis chamber wells and **(A)** tadpole or **(B)** adult frog granulocytes (10^5^ cells/well) were loaded into top wells, separated by 5 μm pore filters. After 3 h of incubation, the filters were stained with Giemsa, mounted bottom side up and the numbers of migrating cells per field of view enumerated. Cells from three individual animals were used for each chemokine concentration (*N* = 3) and the highest tadpole (10^−4^ ng/ml of rCXCL8a; 10^1^ ng/ml of rCXCL8b) and adult frog (10^−6^ ng/ml of rCXCL8a; 10^−4^ ng/ml of rCXCL8b) granulocyte chemotactic activities were confirmed in two additional, independent experiments and the results combined and presented here (*N* = 9). Chemokinesis was measured by adding respective concentrations of rCXCL8 or rCXCL8b that elicited maximal cell migration, to both lower and upper chambers. **(C)** Tadpole and adult frog gene expression of *Cxcr1* and *Cxcr2* relative to *Gapdh*. All results are presented as means + SEM. Above-head letters denote statistical designations: experimental groups described by distinct letters are statistically different (*P* < 0.05) while those marked by the same letters are not.

The distinct *Cxcl8a* and *Cxcl8b* gene expression patterns suggested that these two chemokine isoforms could have non-overlapping functional roles. To discern this possibility, we produced both CXCL8a and CXCL8b in recombinant form (rCXCL8a and rCXCL8b) and examined the dose-dependent capacities of these respective proteins to chemoattract tadpole and adult frog granulocytes (Figures 4A,B, respectively), using blind well chemotaxis chambers. While both rCXCL8a and rCXCL8b elicited characteristic bell shaped dose-dependent chemotaxis of tadpole granulocytes, the rCXCL8b-induced chemotaxis peaked at higher concentrations of the recombinant chemokine (10^1^ ng/mL) and decreased at subsequently lower doses (Figure 4A). By contrast, the rCXCL8a-mediated tadpole granulocyte chemotaxis peaked at much lower concentration (10^−4^ ng/mL; Figure 4A). Similarly, adult frog granulocyte chemotaxis toward rCXCL8a peaked at a higher concentration of the chemokine (10^−6^ ng/ml) than chemotaxis toward rCXCL8b (10^−4^ ng/ml; Figure 4B), together indicating that CXCL8b is a more potent chemoattractant of both tadpole and adult granulocytes than CXCL8a.

To confirm that the observed cell migration was gradient dependent (chemotaxis) rather than increased random cell motility (chemokinesis), we performed chemokinesis experiments using tadpole and adult granulocytes. To this end, we abolished rCXCL8a and rCXCL8b gradients by adding the respective optimal doses of either chemokine to both upper and lower chemotaxis chambers and measured tadpole and adult frog granulocyte migration (Figures 4A,B). Tadpole granulocyte chemotaxis toward rCXCL8a and rCXCL8b, and the adult frog granulocyte chemotaxis toward rCXCL8a (but not to rCXCL8b) was substantially reduced, but not completely abolished under these conditions, indicating that some of the observed granulocyte migration was due to chemokinesis (Figure 4A,B). Conversely, the adult frog granulocyte migration toward rCXCL8b was abolished in the chemokinesis experiments, indicating that this migration was entirely gradient dependent chemotaxis and not chemokinesis (Figure 4B).

Because the tadpole and adult frog granulocytes differed in their chemotactic activity toward the rCXCL8a and rCXCL8b (Figures 4A,B), we examined their gene expression of the putative CXCL8 receptors, *Cxcr1* and *Cxcr2* (30) (Figure 4C). The tadpole G-CSF-granulocytes exhibited greater expression of both *Cxcr1* and *Cxcr2* genes than the adult G-CSF granulocytes (Figure 4C). Interestingly, while the tadpole granulocytes exhibited significantly greater transcript levels of *Cxcr2* than *Cxcr1*, the adult frog granulocytes possessed similar mRNA levels for both receptors (Figure 4C).

### The frog CXCL8a and CXCL8b signal through CXCR1 and CXCR2

**Figure 5:**
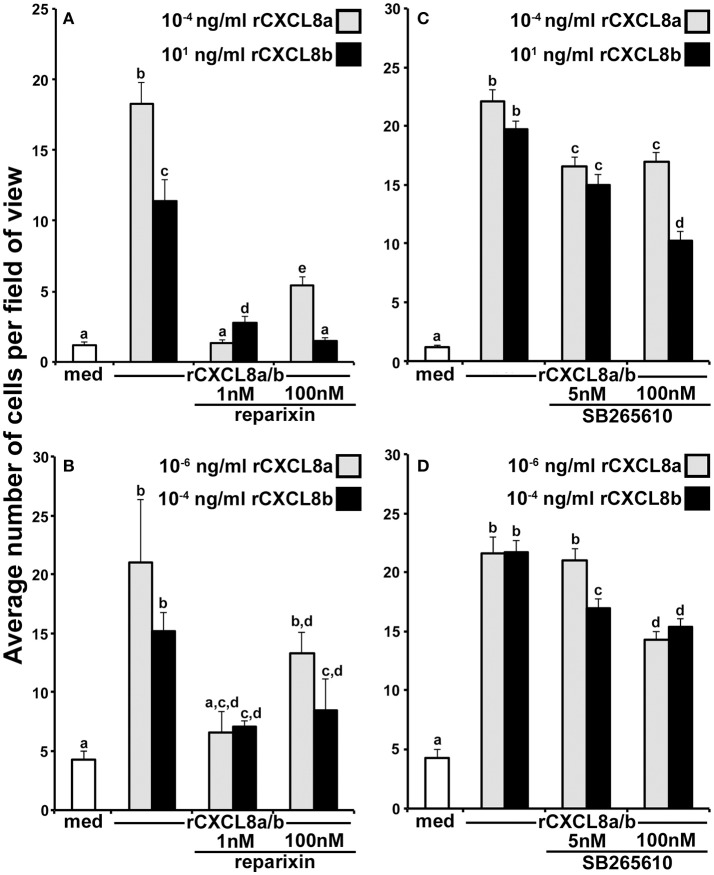
Roles of the frog CXCR1 and CXCR2 in the rCXCL8a- and rCXCL8b-elicited chemotaxis**. (A)** Tadpole and **(B)** adult frog granulocytes (10^5^ cells/well) were subjected to chemotaxis assays in absence or presence of 1 nM or 100 nM of the CXCR1/CXCR2 inhibitor, reparixin and using the respectively most chemo-attractive concentrations of rCXCL8 or rCXCL8b. Cells from three individual animals were used for each chemokine concentration (*N* = 3). **(C)** Tadpole and **(D)** adult frog granulocytes (10^5^ cells/well) were subjected to chemotaxis assays in absence or presence of 5 or 100 nM of the CXCR2 inhibitor, SB265610 and using the respectively most chemo-attractive doses of rCXCL8 or rCXCL8b. Cells from four individual animals were used for each chemokine concentration (*N* = 4). All results are presented as means + SEM. Above-head letters denote statistical designations: experimental groups described by distinct letters are statistically different (*P* < 0.05) while those marked by the same letters are not.

The mammalian CXCR1 and CXCR2 receptors may be pharmacologically inhibited by 1 and 100 nM of reparixin, respectively (30). To discern whether the tadpole and adult frog granulocyte chemotaxis to rCXCL8a and rCXCL8b was mediated by CXCR1 and/or CXCR2, we performed chemotaxis experiments using optimal concentrations of the respective chemokines in the absence or presence of 1 or 100 nM of reparixin (Figures 5A,B). At 1 nM, reparixin reduced the tadpole and adult frog granulocyte chemotaxis toward rCXCL8a to background levels and significantly reduces these cells' migration toward rCXCL8b, albeit not to background levels (Figures 5A,B). The 100 nM reparixin treatment was less effective at inhibiting the tadpole and adult frog granulocyte chemotaxis toward rCXCL8a, abolished the tadpole cell migration toward rCXCL8b to background levels, and decreased the adult frog granulocyte migration toward rCXCL8b to levels comparable to those seen at the 1 nM dose of the antagonist (Figures 5A,B).

To reevaluate the roles of CXCR2 in the rCXCL8a and rCXCL8b chemotaxis of tadpole and adult frog granulocytes, we repeated the above experiments, this time utilizing a specific CXCR2 inhibitor, SB265610 (Figures 5C,D). This compound inhibits the mammalian neutrophil chemoattractant-induced calcium mobilization and neutrophil chemotaxis at 3.7 and 70 nM, respectively (31). Accordingly, we used final SB265610 concentrations of 5 and 100 nM for our tadpole and adult frog granulocyte CXCR2 inhibition assays. At 5 nM, SB265610 had a modest but significant inhibitory effect on the tadpole granulocyte chemotaxis toward rCXCL8a and rCXCL8b (Figure 5C). The 100 nM dose of SB265610 did not further decrease the tadpole granulocyte chemotaxis toward rCXCL8a (Figure 5C). Interestingly, the 100 nM dose of SB265610 resulted in significantly greater inhibition of the rCXCL8b-mediated chemotaxis than the inhibition of the rCXCL8a-mediated migration (Figure 5C). Concurrently, this inhibition of rCXCL8b-mediated activity was significantly greater than that seen at 5 nM of the compound (Figure 5C).

The 5 nM dose of SB265610 resulted in modest, but significant inhibition of the rCXCL8b-, but not the rCXCL8a-mediated chemotaxis of adult frog granulocytes (Figure 5D). Conversely, at the 100 nM, this inhibitor resulted in comparable significant abrogation of the adult frog granulocyte chemotaxis toward both rCXCL8a and rCXCL8b, (Figure 5D).

### The tadpole CXCL8a and CXCL8b chemoattract distinct granulocyte populations

**Figure 6:**
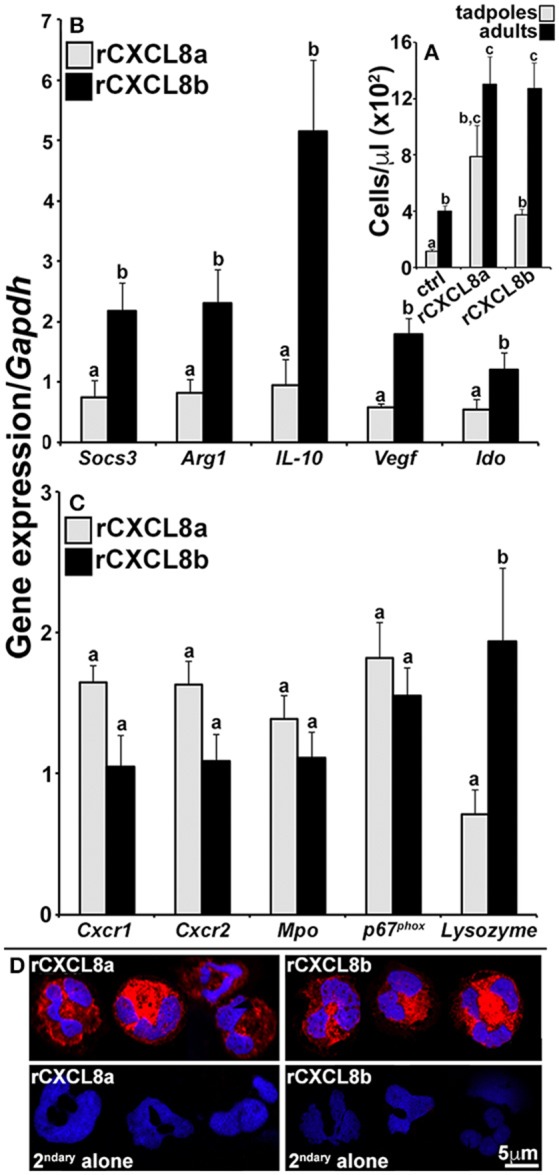
Analysis of immune gene expression and cytology of rCXCL8a- and rCXCL8b-elicited tadpole cells. Tadpoles and adult frogs were injected intraperitoneally with rCXCL8a or rCXCL8b (1 μg/g of body weight) in 10 μl of saline or with an equal volume of the vector control (supernatants from empty vector-transfected Sf9II cell, processed in parallel to rCXCL8a and rCXCL8b production). After 4 h, animals were lavaged with saline and the cells were enumerated **(A)**. The tadpole **(B,C)** and adult frog (not presented) rCXCL8a and rCXCL8b-elicited cell immune gene expression was examined relative to the *Gapdh* control (*N* = 6). All results are presented as means + SEM. Above-head letters denote statistical designations: experimental groups described by distinct letters are statistically different (*P* < 0.05) while those marked by the same letters are not. **(D)** Tadpole rCXCL8a and rCXCL8b-elicited cells were stained with a rabbit anti-frog rG-CSFR (or saline) Ab and secondary goat anti-rabbit Ab and examined by confocal microscopy.

In consideration of the potentially disparate roles of the frog CXCL8a and CXCL8b, we intraperitoneally administered rCXCL8a and rCXCL8b to tadpoles and adult frogs, recovered the resulting peritoneal exudates, and examined these cells for their expression of a panel of immune genes (Figure 6). The rCXCL8a and rCXCL8b elicited similar numbers of adult cells bearing granulocyte morphology whereas rCXCL8a chemoattracted more cells into tadpole peritonea than rCXCL8b (Figure 6A). The adult rCXCL8a- and rCXCL8b-elicited cells did not exhibit significant transcriptional differences (data not shown). The tadpole rCXCL8a and rCXCL8b-chemoattracted cells also expressed comparable levels of *Cxcr1, Cxcr2*, NADPH oxidase catalytic subunit *p67phox* and myeloperoxidase (*Mpo;* Figure 6C). Conversely and in comparison to rCXCL8a-recruited cells, the rCXCL8b-elicited population possessed significantly greater mRNA levels of *lysozyme* (Figure 6C) as well as genes associated with immune suppression and wound repair, including suppressor of cytokine signaling 3 (*Socs3*), arginase-1 (*Arg1*), interleukin-10 (*IL-10*), vascular endothelial growth factor (*Vegf*), and indoleamine 2,3 dioxygenase (*Ido*; Figure 6B).

While our results indicated that the tadpole G-CSF granulocytes express greater levels of *Cxcr2* than C*xcr1* (Figure 4C), we did not see such expression patterns in the tadpole rCXCL8a- and rCXCL8b-elicited cells (Supplemental Figure 2B). Moreover, while the tadpole G-CSF granulocytes expressed greater levels of both of the *Cxcr1* and *Cxcr2* genes than the adult G-CSF granulocytes (Figure 4C), in fact the adult frog rCXCL8a- and rCXCL8b-elicited cells expressed greater levels of both receptors than their tadpole counterparts (Supplemental Figure 2B).

To confirm that the rCXCL8a- and rCXCL8b-recruited cells were in fact granulocytes, we generated a polyclonal antibody against a recombinant form of the frog G-CSFR and stained tadpole chemokine-derived cells with this reagent. Both rCXCL8a- and rCXCL8b-elicited exudates were predominantly composed of G-CSFR-positive cells bearing characteristic polymorphonuclear granulocyte morphology (Figure 6D).

## Discussion

Vertebrate chemokine genes are believed to have diverged more rapidly with evolutionary time than most other components of the vertebrate immune system (32, 33), presumably reflecting the distinct physiological and evolutionary pressures of these diverging species. This notion is largely supported by the vast and highly distinct chemokine ligand and receptor repertoires seen across different species (34). It is interesting to consider that despite this apparent diverging evolutionary pressure on the vertebrate chemokine genes, interleukin-8 and its cognate receptors (CXCR1 and CXCR2) are retained in most of these species, albeit in multiple isoforms across some. This suggests that CXCL8 plays important biological roles that cannot be as easily amended to the evolutionary pressures faced with species' divergence. Conversely, the presence of multiple CXCL8 isoforms presumably permits the neo-functionalization of CXCL8 isoforms without compromising the indispensable, evolutionarily conserved roles of CXCL8. This is exemplified in the cyprinid fish CXCL8_L1 and CXCL8_L2 lineages (15, 34) as well as in the *X. laevis* frog CXCL8a and CXCL8b, as presented here. Indeed, the frog CXCL8a and CXCL8b possess fairly distinct protein sequences, suggesting that they have diverged with evolutionary time. In the case of the *X. laevis* CXCL8a and CXCL8b gene expression and function, our results suggest that the frog CXCL8a is serving the inflammatory roles attributed to other vertebrate CXCL8 molecules while the frog CXCL8b may have adopted unique biological roles, at least within the tadpole life of this animal. It is notable that adult frog granulocytes upregulated their *Cxcl8b* gene expression following bacterial and viral stimulation, suggesting that CXCL8b may also play a role during the adult frog immune responses to certain pathogens. Further research will revel the exact role of CXCL8b during frog immune responses.

Here we report that, *Xenopodinae* frogs possess both an ELR motif-containing CXCL8 (CXCL8a) as well as an CXCL8 (CXCL8b) that lacks this motif. Moreover, these chemokine protein sequences are somewhat distinct, supporting the notion that they may have diverged in their respective functions. Indeed, the ELR motif-lacking CXCL8b branches ancestrally to the frog CXCL8a proteins as well as to all higher vertebrate CXCL8s. *Xenopodinae* frogs are presently known to possess three additional putative CXCR1/CXCR2 ligands; CXCL2, CXCL5, CXCL6, of which only the CXCL2 possesses an ELR motif (sequences available on GenBank). By contrast, while chickens also possess three putative CXCR1/CXCR2 ligands, including two CXCL8 isoforms, all of these proteins possess ELR motifs (35). This suggests that emergence of ELR motif-containing CXCL8 proteins as well as other CXCR1/CXCR2 ligands occurred in tetrapods. Despite lacking an ELR motif, the fish CXCL8 proteins are chemoattractive to neutrophils (11, 12), bringing to question the functional significance of the emergence of ELR-bearing CXCL8 chemokines. Further work using animal models like *Xenopus*, which possess both ELR-containing and ELR-lacking CXCL8 chemokines will be invaluable to addressing this question.

Our chemotaxis experiments using recombinant forms of the *X. laevis* CXCL8a and CXCL8b indicate that these proteins have distinct capacities to chemoattract tadpole and adult frog granulocytes. In general, when performing *in vitro* chemotaxis assays, increasing chemokine concentrations results in the loss of the chemokine gradient across the chemotaxis chambers, leading to chemokine receptor saturation and preventing further migration, which results the characteristic bell shape of the dose-response curves (36), akin to those reported here for rCXCL8a and rCXCL8b. In this respect, it is notable that in the case of both tadpole and adult granulocyte chemotaxis, the migration peaked at higher doses of rCXCL8a than rCXCL8b. This indicates that rCXCL8a-mediated receptor saturation occurs at higher doses than with rCXCL8b, in turn suggesting that the receptor-ligand interactions of these two chemokines are distinct.

While 1 and 100 nM concentrations of reparixin block the signaling through the mammalian CXCR1 and CXCR2, respectively (30), our results indicated that the 1 nM reparixin effectively blocked the rCXCL8a- and rCXCL8b-mediated chemotaxis while the 100 nM dose did not confer further inhibitory effects. Notably, our pharmacological inhibition of CXCR2 resulted in decreased tadpole and adult frog granulocyte chemotaxis toward rCXCL8a and rCXCL8b, together indicating that both CXCR1 and CXCR2 are engaged by the CXCL8a and CXCL8b. These results may reflect the fact that the downstream signaling through the frog CXCR1 and CXCR2 is significantly more sensitive to reparixin inhibition than the counterpart mammalian receptors. Interestingly, the tadpole but not the adult frog granulocytes expressed greater levels of *Cxcr2* than *Cxcr1* while the 100 nM dose of the CXCR2 antagonist resulted in significantly greater reduction in tadpole (but not adult frog) granulocyte chemotaxis toward rCXCL8b, than rCXCL8a. Possibly, the frog CXCL8b relies more heavily on signaling through the CXCR2 for the recruitment of tadpole granulocytes. In this respect, we were surprised to find that the tadpole granulocytic cells recruited by rCXCL8a and rCXCL8b did not exhibit the greater *Cxcr2* expression seen in the tadpole G-CSF-granulocytes. Presumably, the G-CSF-granulocytes, the rCXCL8a- and rCXCL8b-elicited granulocytes represent distinct tadpole immune populations and/or differentially activated granulocyte subsets. The mammalian granulocyte gene expression of *Cxcr1* and *Cxcr2* is tenuous and subject to their activation states (37), so it is possible that the downstream signaling elicited by CXCL8b in tadpole granulocytes may be dampening the *Cxcr2* gene expression or enhancing the gene expression of *Cxcr1*. Moreover, *X. laevis* are presently thought to encode single copies of *Cxcr1* and *Cxcr2* genes, ruling out the possibility that CXCL8a and CXCL8b function through distinct receptor isoforms. In consideration of our findings, we speculate that the frog CXCL8a and CXCL8b may have distinct affinities for CXCR1 and CXCR2, culminating in their disparate chemotactic properties. It is also notable that tadpole and adult granulocyte chemotaxis peaked at distinct concentrations of either chemokine, suggesting differences between tadpole and adult responsiveness to CXCL8a and CXCL8b. These differences may be explained by the differences in the *Cxcr1* and *Cxcr2* gene expression by the tadpole and adult frog cells.

Amphibian tadpoles differ from adult frogs in their ability to regenerate amputated limbs (38) and tadpoles experience a refractory period during which they temporarily lose this regenerative capacity (39). Interestingly, this refractory period may be circumvented by treating tadpoles with certain anti-inflammatory, immunosuppressive, or antioxidant agents (39). This is particularly notable considering that our results indicate that tadpole Cxcl8b gene expression is increased during wound repair while tadpole (but not adult) rCXCL8b-recruited granulocytes express immunosuppressive and anti-inflammatory genes (IL-10, Socs3, Ido). Indeed, IL-10 is a hallmark anti-inflammatory mediator (40), SOCS3 is associated with immunosuppressive immune states (41), and the IDO enzyme is associated with immune modulation and the induction of immunological tolerance (42). Moreover, the tadpole rCXCL8b-elicited granulocytes also exhibited elevated expression of vascular endothelial growth factor and arginase-1, which are very important in angiogenesis (43) and to tissue repair (44), respectively. Together, our findings strongly suggest that in tadpoles, CXCL8b is involved in recruiting a subset of granulocytes that are functionally polarized akin to alternatively-polarized (M2) macrophages (45), as immunosuppressive, healing effectors that may be playing a role during tadpole wound-healing and tissue regeneration.

The duplication of vertebrate genes and their subsequent neo-functionalization is widely believed to being a major driving force behind speciation and species-specific physiological diversification (46). Considering the unique physiological and pathogenic pressures that have molded amphibian physiology, together with their highly distinct tadpole and adult life stages; it is intuitive that they would have developed distinct mechanisms for dealing with their physiological demands. Amphibian tadpole development is closely linked to their temporal regulation of inflammatory genes (47) and it is intriguing to consider that their use of CXCL8b as a means for immune suppression and tissue repair may have evolved out of the inflammatory functions associated with CXCL8.

## Ethics statement

This study was carried out in accordance with the recommendations of and following approval by the Institutional Animal Care and Use Committee (approval number 15-024).

## Author contributions

DK and LG designed and planned the studies. DK, AY, and MP performed the experiments. DK and LG analyzed the data, wrote the manuscript, and prepared the figures.

### Conflict of interest statement

The authors declare that the research was conducted in the absence of any commercial or financial relationships that could be construed as a potential conflict of interest.

## Supplementary material

Supplemental Figure 1 Western blot of the produced, purified, and eluted rCXCL8a and rCXCL8b fractions. The eluted recombinant (r)CXCL8a and rCXCL8b were resolved by SDS PAGE, transferred onto nitrocellulose membranes and western blots were performed using an HRP-conjugated mouse anti-V5 (Sigma) to determine which elution fractions contained rCXCL8a (15 kDa) and rCXCL8b (16 kDa).

Supplemental Figure 2 Analyses of the anti-G-CSFR polyclonal antibody and the rCXCL8a- and rCXCL8b-elicited granulocyte expression of the *Cxcr1* and *Cxcr2* genes. **(A)** Rabbits were immunized with rG-CSFR and the resulting serum was applied to a HiTrap Proetin A HP column (GE Health) to isolate the IgG fraction, and to a rG-CSFR-bound Sulfo-Link Protein column to purify the IgG fraction that cross-reacted with the rG-CSFR. To confirm the specificity of this reagent, this anti-rG-CSFR IgG farction was used to perform a western blot of the rG-CSFR before (lane 1) or after (lane 2) pre-absorbing the Ab against the rG-CSFR. **(B)** Tadpoles and adult frogs were injected intraperitoneally with rCXCL8a or rCXCL8b (1 μg/g of body weight) in 10 μl of saline or with an equal volume of the vector control. After 4 h, animals were lavaged with saline and the cells were enumerated and examined for their expression of *Cxcr1* and *Cxcr2*. Above-head letters denote statistical designations: experimental groups described by distinct letters are statistically different (*P* < 0.05) while those marked by the same letters are not.
