# Supplementary figures and images for: Amphibian (Xenopus laevis) Interleukin-8 (CXCL8): A Perspective on the Evolutionary Divergence of Granulocyte Chemotaxis
Source: Front Immunol. 2018 Sep 12;9:2058. doi: 10.3389/fimmu.2018.02058 (PMC6145007; doi:10.3389/fimmu.2018.02058)

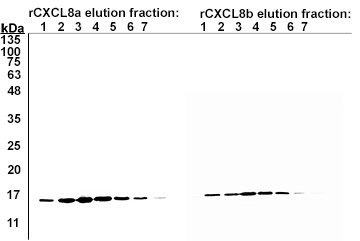

Supplement: Supplemental Figure 1 — Western blot of the produced, purified, and eluted rCXCL8a and rCXCL8b fractions. The eluted recombinant (r)CXCL8a and rCXCL8b were resolved by SDS PAGE, transferred onto nitrocellulose membranes and western blots were performed using an HRP-conjugated mouse anti-V5 (Sigma) to determine which elution fractions contained rCXCL8a (15 kDa) and rCXCL8b (16 kDa). [file Image_1.TIF]

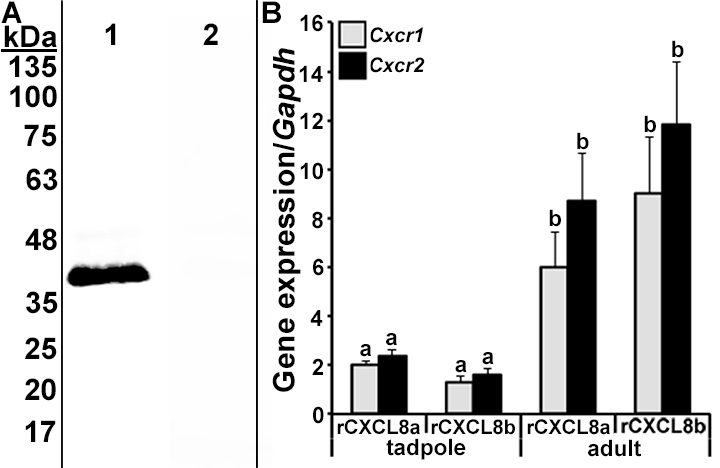

Supplement: Supplemental Figure 2 — Analyses of the anti-G-CSFR polyclonal antibody and the rCXCL8a- and rCXCL8b-elicited granulocyte expression of the Cxcr1 and Cxcr2 genes. (A) Rabbits were immunized with rG-CSFR and the resulting serum was applied to a HiTrap Proetin A HP column (GE Health) to isolate the IgG fraction, and to a rG-CSFR-bound Sulfo-Link Protein column to purify the IgG fraction that cross-reacted with the rG-CSFR. To confirm the specificity of this reagent, this anti-rG-CSFR IgG farction was used to perform a western blot of the rG-CSFR before (lane 1) or after (lane 2) pre-absorbing the Ab against the rG-CSFR. (B) Tadpoles and adult frogs were injected intraperitoneally with rCXCL8a or rCXCL8b (1 μg/g of body weight) in 10 μl of saline or with an equal volume of the vector control. After 4 h, animals were lavaged with saline and the cells were enumerated and examined for their expression of Cxcr1 and Cxcr2. Above-head letters denote statistical designations: experimental groups described by distinct letters are statistically different (P < 0.05) while those marked by the same letters are not. [file Image_2.TIF]
