# Supplementary material for: Amphibian (Xenopus laevis) Interleukin-8 (CXCL8): A Perspective on the Evolutionary Divergence of Granulocyte Chemotaxis
Source: Front Immunol. 2018 Sep 12;9:2058. doi: 10.3389/fimmu.2018.02058 (PMC6145007; doi:10.3389/fimmu.2018.02058)
Supplement: Supplementary file 3 [file Table_1.docx]

**Supplemental table 1**

List of primer sequences

| **Primer** | **Sequence (5’-3’ forward, reverse)** |
| --- | --- |

| ***Arg1*** | TCCAAGGGACAGCCAAGAAG |
| --- | --- |
|  | CTCGAACATCATTGCCAAATTC |
| ***Cxcr1*** | CCAGTGGGTGTTTGGAATCT |
|  | GCAAGGTATCGGTCAACACTAA |
| ***Cxcr2*** | AGGATAGGGAGACACTTGGTAG |
|  | GGCTGCTCTTGGTTTGATAGA |
| ***Gapdh*** | ACCCCTTCATCGACTTGGAC |
|  | AGATGGAGGAGTGAGTGTCACCAT |
| ***Gcsfr*** | TGGATGAAGGACTACAGCTAATG |
|  | GCCTGTCATCTGTGAGGTTTA |
| ***Ido*** | TATTGCGGATGCGAGAGTACA |
|  | TCCCCGCTTTCTTGAACGTA |
| ***IL-8a*** | CCAGTGTCAAGGACCGG |
|  | CCCACTTGTCAAAGTTGC |
| ***IL-8b*** | TCTAGAAGTCATAATCACAATTAA |
|  | ATCTGTTTGAGTCACTGGCTC |
| ***IL-10*** | TGCTGGATCTTAAGCACACCCTGA |
|  | TGTACAGGCCTTGTTCACGCATCT |
| ***Lysozyme*** | GCACAGCTTTCTTTGAGAGTTC |
|  | TACACCACCAACGACTGTTTA |
| ***Mpo*** | CCAGAACCGAAGTGACTGTATC |
|  | TGGCATCAACATAAGAGGTGAGA |
| ***Socs3*** | GCTCCAAACGGGCATATTA |
|  | CTACATAGGTGCTGGAGAGA |
| ***Vegf*** | CCAGGAGAGGGAGACCATAAA |
|  | CCTGGAATATATCCACCAGAATCTC |
